# Axillary response and outcome in breast cancer patients after neoadjuvant treatment: The role of radiotherapy in reducing recurrence in ypN0 patients with initially cN+ stage

**DOI:** 10.3389/fonc.2023.1093155

**Published:** 2023-04-03

**Authors:** Xiaoqiu Ren, Yaner Yu, Lihong Liu, Wenjie Xia, Runliang Ni, Shumei Wei, Jun Wu, Qichun Wei

**Affiliations:** ^1^ Department of Radiation Oncology, Key Laboratory of Cancer Prevention and Intervention, Ministry of Education, Second Affiliated Hospital, Zhejiang University School of Medicine, Hangzhou, Zhejiang, China; ^2^ General Surgery, Cancer Center, Department of Breast Surgery, Zhejiang Provincial People’s Hospital (Affiliated People’s Hospital, Hangzhou Medical College), Hangzhou, Zhejiang, China; ^3^ Department of Pathology, Second Affiliated Hospital, Zhejiang University School of Medicine, Hangzhou, Zhejiang, China

**Keywords:** breast cancer, neoadjuvant treatment, radiation, lymph node, prognosis

## Abstract

**Objective:**

We aim to explore the clinicopathological features associated with axillary node response and recurrence in breast cancer patients undergoing neoadjuvant treatment (NAT).

**Methods:**

We retrospectively reviewed the medical records of 486 stage I to III breast cancer patients who received NAT and surgery between 2016 and 2021.

**Results:**

A total of 486 cases were reviewed and 154 (31.7%) patients achieved breast pathological complete response (pCR) (ypT0/Tis). Of the 366 cases with initially cN+, 177 (48.4%) cases reach ypN0. Breast pCR is in high accordance to axillary pCR (81.5%). Hormone receptor (HR)-/HER2+ breast cancer patients have the highest axillary pCR rate (78.3%). Patients achieve axillary pCR have a significantly better disease-free survival (DFS) (P=0.0004). Further analysis reveals that the DFS of ypN0 and ypN1 cases are similar (*P*=0.9049). Moreover, DFS in patients with ypN0 (*P*<0.0001) and ypN1 (*P*<0.0001) is significantly better than that in patients with ypN2-3. For post-mastectomy ypN0 cases, radiation could only improve DFS in patients with initially cN+ stage (*P*=0.0499). Multivariate Cox regression analysis shows that radiation is an independent factor to improve DFS (Hazard ratio (HR): 0.288(0.098-0.841), *P*=0.0230). Radiation does not improve DFS in pre-cN0/ypN0 patients (*P*=0.1696).

**Conclusion:**

Axillary pCR rate is higher than breast pCR rate. HR-/HER2+ patients have the highest axillary pCR rate. Axillary pCR is associated with better DFS. Radiation could further improve DFS in ypN0 patients with initially positive nodal disease.

## Introduction

Neoadjuvant treatment (NAT) has widely been used in the treatment of early-stage breast cancer. Pathological complete response (pCR), usually defined as ypT0/Tis with or without ypN0, is not the only aim of NAT but undeniably an outstanding marker for predicting prognosis (1). It has been reported that nodal status after NAT but not initially N stage was significantly associated with survival. Axillary pCR is a potential prognostic indicator of better 5-year overall survival (OS) (2). Breast pCR is highly consistent with axillary pCR, especially in HER2+ and triple negative breast cancer (TNBC) (3, 4). The HER2+ breast cancer and TNBC were also reported to have the highest axillary pCR rate (5). In these two subtypes of breast cancer, the risk of residual nodal metastasis in breast pCR and cN0/ycN0 patients is quite low. Therefore, it may be possible to avoid axillary lymph node dissection (ALND) (3, 6). However, axillary pCR is only a moderate predictor of breast pCR, indicating that the response of breast and axillary to NAT is not identical (4).

The pre- and post-NAT nodal stages are important considerations for the use adjuvant radiation treatment (RT). However, it is still various for clinical practice of adjuvant RT in patients with ypN0 (7, 8). In all breast cancer patients after NAT, the use of adjuvant RT is associated with a trend of lower local relapse (8). Data from National Cancer Data Base (NCDB) indicated that adjuvant RT conferred significant overall survival (OS) improvement in ypN1 patients. For ypN0 cases, RT was only associated with OS benefits in post-lumpectomy patients, but not in post-mastectomy ones (7). However, other studies have reported that adjuvant RT could improve OS in all ypN stages subgroups after mastectomy (9). Moreover, the regional nodal irradiation shows no extra survival improvement compared with breast-only RT (7, 9, 10).

We aim to explore the clinicopathological features associated with axillary response and prognosis of post-NAT patients with different ypN stages. We also aim to investigate patients who benefited from post-mastectomy RT (PMRT) most. Here, we present our findings.

## Materials and methods

### Patients

The data of female breast cancer patients who received NAT and surgery in the Second Affiliated Hospital of Zhejiang University (SAHZU) and Zhejiang Provincial People’s Hospital (ZPPH) from 2016 to 2021 were retrospectively reviewed. The inclusion criteria are as follows: (1) biopsy was performed before treatment, and breast cancer was confirmed by histopathology; (2) patients underwent NAT and post NAT surgery in these two medical centers; (3) follow-up time was longer than one month. Exclusion criteria included: (1) patients initially diagnosed as stage IV; (2) lack of necessary data in medical records; (3) bilateral breast cancer. This study was approved by the Ethics Committee of SAHZU and ZPPH. The study was carried out in accordance with the Declaration of Helsinki.

### Statistical analysis

Disease-free survival (DFS) was calculated from the date of surgery to the first local or distant recurrence, death or last follow-up without recurrence. The differences of distribution were analyzed using χ2 or Fisher exact test. A modified Poisson regression model was used to estimate the relative risk between factors and axillary pCR rate. The survival curves were drawn by Kaplan–Meier method. Log-rank P was calculated to compare the differences between survival curves. The hazard ratio (HR) and 95% confidence interval (CI) were calculated using the univariable and multivariable Cox models. The figures were drawn using Graph Prism 9.3.0 and analyses were performed using IBM SPSS for Windows version 26.0. P<0.05 was considered statistically significant.

## Results

A total of 486 cases were included in this study. The median age of the cohort was 51 years (24-75 years). The median follow-up time was 25 months (range, 1-70 months). According to immunohistochemistry (IHC) and fluorescence *in situ* hybridization (FISH) results of breast and lymph node biopsy specimen, 176 (36.2%) cases were categorized as hormone receptor (HR)+/HER2- (Luminal A) type. 140 (28.8%) cases were HR+/HER2- (Luminal B). 80 (16.5%) cases were HER2 amplified and 90 (18.5%) cases were TNBC. Most patients (n=337, 70.4%) were staged as initially cT2 and cT3-4 cases (n=72) accounted for only 15.0%. T stages were unknown in 7 cases. 366 cases (75.3%) were initially cN+ (325 cases were pathologically proven) and 120 cases (24.7%) were initially cN0 (70 cases were pathologically proven). In our cohort, 372 cases (76.5%) underwent mastectomy and 433 cases (89.1%) underwent axillary lymph node dissection (ALND). In total, 154 (31.7%) patients achieved breast pCR (ypT0/Tis) and 269 (55.3%) patients achieved axillary pCR (ypN0). Characteristics of cohort are summarized in **Table 1**.

**Table 1:** Baseline characteristics of population.

Characteristics	n (%)	Characteristics	n (%)
No. of patients	486	Mean age	50.9 (24-75)
ER status		pre-cN	
Positive	286 (58.8)	cN0	120 (24.7)
Negative	200 (41.2)	cN+	366 (75.3)
PR status		Breast response	
Positive	209 (43.0)	MP1	11 (22.3)
Negative	277 (57.0)	MP2	103 (21.2)
HER2 status		MP3	146 (30.0)
Positive	220 (45.3)	MP4	72 (14.8)
Negative	266 (54.7)	MP5	154 (31.7)
Ki-67		Axillary response	
≤15%	106 (21.8)	ypN0	269 (55.3)
>15%	380 (78.2)	ypN+	217 (44.7)
Subtypes		Histology	
HR+HER2- (Luminal A)	176 (36.2)	Ductal	438 (90.1)
HR+HER2+ (Luminal B)	140 (28.8)	Lobular	11 (2.3)
HER2 amplified	80 (16.5)	Mucous	6 (1.2)
TNBC	90 (18.5)	Micropapillary	19 (3.9)
AR status		Others	12 (2.5)
Positive	211 (85.1)	Breast surgery	
Negative	37 (14.9)	Mastectomy	372 (76.5)
Unknown	238	Breast conserving	114 (23.5)
Grade		Axillary surgery	
I	30 (12.0)	SLNB	53 (10.9)
II	159 (63.9)	ALND	422 (86.8)
III	60 (24.1)	SLNB+ALND	11 (2.3)
Unknown	237	Neoadjuvant regimens	
pre-cT		Anthracycline containing	386 (79.4)
0	2(0.4)	Taxane containing	452 (93.0)
1	68(14.2)	Single HER2 blockade	151 (31.1)
2	337(70.4)	Dual HER2 blockade	39 (8.0)
3	67(14.0)	Radiation therapy	
4	5(1.0)	Yes	336 (69.1)
Unknown	7	No	150 (30.9)

ER, estrogen receptor; PR, progesterone receptor; HER2, human epidermal growth factor receptor2; HR, hormone receptor; TNBC, triple negative breast cancer; AR, androgen receptor; pre-cT, clinical tumor stage before treatment; pre-cN, clinical nodal stage before treatment; MP, Miller-Payne; SLNB, sentinel lymph node biopsy; ALND, axillary lymph node dissection.

**Figure 1:**
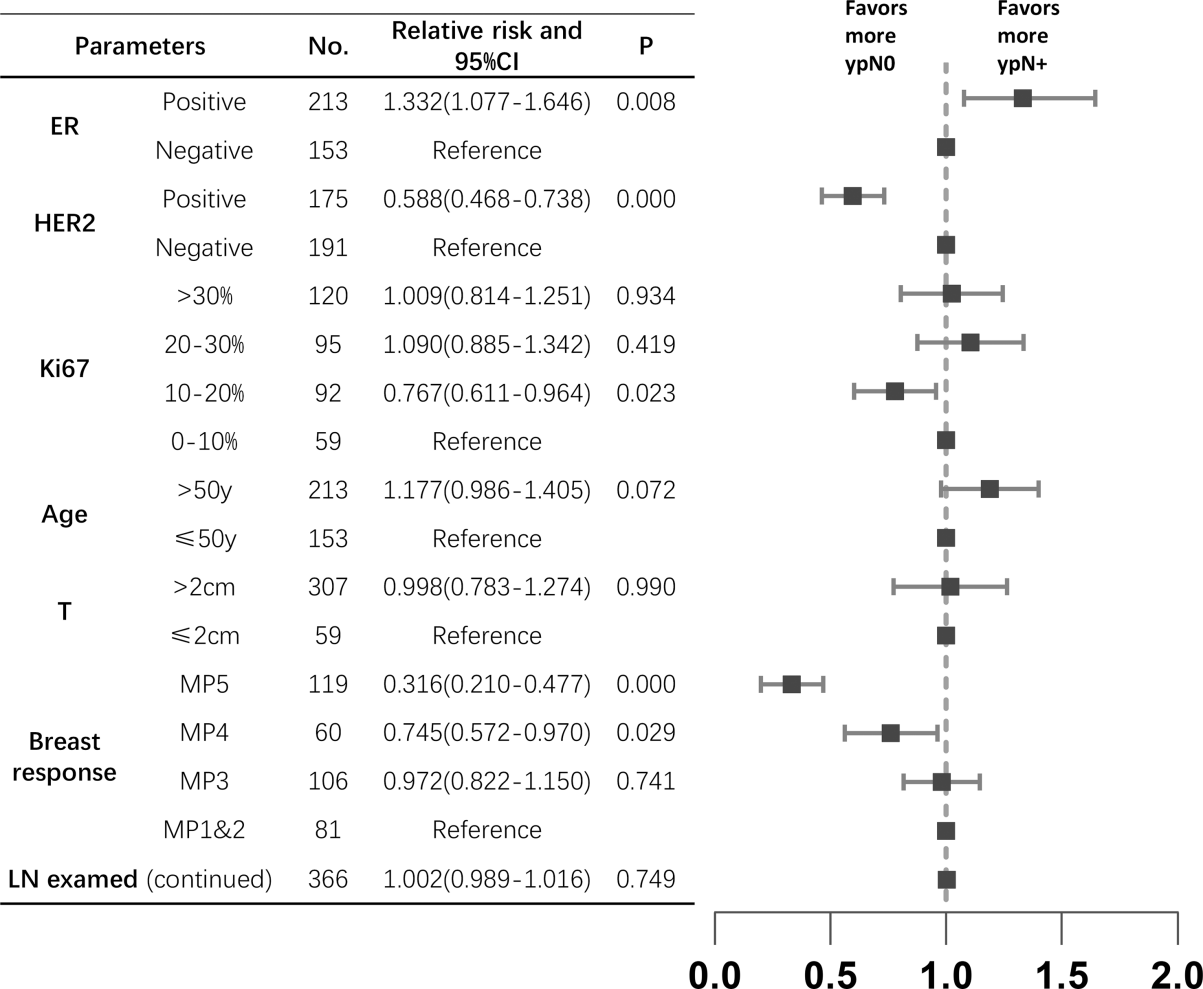
The relative risks between clinicopathological characteristics and axillary response in pre-N+ patients (n=366). ER, estrogen receptor; HER2, human epidermal growth factor receptor2; MP, Miller-Payne; LN, lymph node; CI, confidence interval.

Of the 366 pre-cN+ cases, 177 (48.4%) achieved axillary pCR. However, 28 pre-cN0 cases (28/120, 23.3%) turned out to be ypN+. The luminal B (63.2%) and HER2 amplified (78.3%) patients had the highest axillary pCR rate. For all HER2 positive patients, the axillary response rate was 69.1% (vs HER2-: 29.3%, P<0.0001). Estrogen receptor (ER)- (P<0.0001), progesterone receptor (PR) - (P=0.0002) and Ki67>15% (P=0.019) of primary lesions were associated with higher axillary pCR rate (**Table 2**). The result of Poisson regression analysis indicated that ER positive patients were less likely to achieve axillary pCR (Relative risk (RR) and 95%CI: 1.332(1.077-1.646), P=0.008). HER2 positive patients were more likely to be ypN0 (RR and 95%CI: 0.588(0.468-0.738), P=0.000). Patients with Ki67 10-20%+ had the higher axillary pCR rate than Ki67 0-10%+ group (RR and 95%CI: 0.767(0.611-0.964), P=0.023). The patients with Miller-Payne (MP) grade 5 in breast were significantly likely to be ypN0 (RR and 95%CI: 0.316(0.210-0.477), P=0.000), followed with MP4 patients (RR and 95%CI: 0.745(0.572-0.970), P=0.029) (**Figure 1**).

**Table 2:** The correlation between ypN stages after NAT and clinicopathological characteristics in pre-cN+ patients (n=366).

Parameters	ypN0	ypN+	P
pre-cN (n=486)			0.000
cN+	177(48.4)	189(51.6)	
cN0	92(76.7)	28(23.3)	
ER status			0.000
Positive	80(37.6)	133(62.4)	
Negative	97(63.4)	56(36.6)	
PR Status			0.000
Positive	80(39.6)	122(60.4)	
Negative	97(59.1)	67(40.9)	
HER2 status			0.000
Positive	121(69.1)	54(30.9)	
Negative	56(29.3)	135(70.7)	
Subtypes			0.000
HR+HER2- (Luminal A)	28(21.7)	101(78.3)	
HR+HER2+ (Luminal B)	67(63.2)	39(36.8)	
HER2 amplified	54(78.3)	15(21.7)	
TNBC	28(45.2)	34(54.8)	
Age			0.137
≤50y	81(52.9)	72(47.1)	
>50y	96(45.1)	117(54.9)	
Ki-67			0.019
≤15%	29(36.7)	50(63.3)	
>15%	148(51.6)	139(48.4)	
Grade			0.867
1	7(33.3)	14(66.7)	
2	39(32.2)	82(67.8)	
3	13(28.3)	33(71.7)	
Unknown	118	60	
pre-cT			0.726
0-1	24(50.0)	24(50.0)	
2	126(48.6)	133(51.4)	
3-4	21(42.9)	28(57.1)	
Unknown	6	4	
ypT			0.000
ypT0/Tis	97(81.5)	22(18.5)	
ypT1-4	177(48.4)	189(51.6)	

ER, estrogen receptor; PR, progesterone receptor; HER2, human epidermal growth factor receptor2; HR, hormone receptor; TNBC, triple negative breast cancer; pre-cT, clinical tumor stage before treatment; pre-cN, clinical nodal stage before treatment.

**Figure 2:**
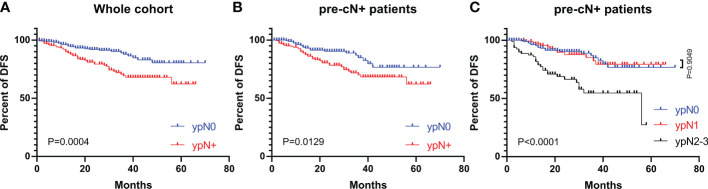
The DFS in patients with different ypN stages from the whole cohort and pre-cN+ groups. **(A)** The DFS of patients with ypN0 and ypN+ stages from the whole cohort (n=486); **(B)** The DFS of patients with pre-cN+/ypN0 and pre-cN+/ypN+ stages (n=366); **(C)** The DFS of patients with ypN0, ypN1 and ypN2-3 stages from pre-cN+ group (n=366).

**Figure 3:**
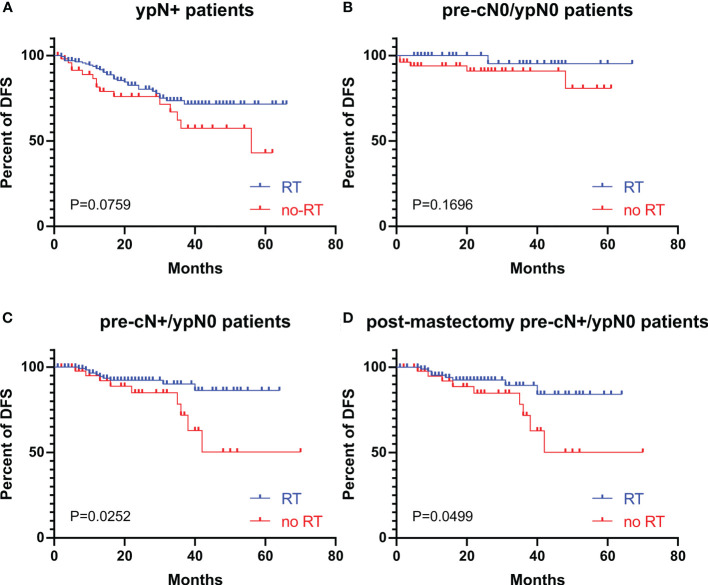
The DFS in patients with or without adjuvant RT with different pre-cN and ypN stages. **(A)** The association between adjuvant RT and DFS in ypN+ patients (n=217); **(B)** The association between adjuvant RT and DFS in pre-cN0/ypN0 patients (n=92); **(C)** The association between adjuvant RT and DFS in pre-cN+/ypN0 patients (n=177); **(D)** The association between adjuvant RT and DFS in post-mastectomy pre-cN+/ypN0 patients (n=142). RT, radiation treatment.

The DFS curves of patients with different ypN stages are shown in **Figure 2**. The ypN0 patients had significantly better DFS than ypN+ patients in both whole cohort (P=0.0004, **Figure 2A**) and pre-cN+ group (P=0.0129, **Figure 2B**). Further analysis revealed that the DFS of ypN0 and ypN1 patients were similar (P=0.9049, **Figure 2C**). However, DFS was significantly better in patients with ypN0 (P<0.0001) and ypN1 (P<0.0001) than in patients with ypN2-3 (**Figure 2C**). Adjuvant RT was associated with improved DFS in pre-cN+/ypN0 (P=0.0252, **Figure 3C**) and post-mastectomy pre-cN+/ypN0 patients (P=0.0499, **Figure 3D**). Multivariate Cox regression analysis showed that PMRT was an independent factor to improve DFS (HR: 0.288(0.098-0.841), P=0.0230, **Table 3**) in patients with pre-cN+/ypN0. RT also showed a tendency to improve DFS in ypN+ patients (P=0.0759, **Figure 3A**). However, adjuvant RT did not benefit DFS in preN0/ypN0 patients (P=0.1696, **Figure 3B**).

**Table 3:** Results of the univariate and multivariate analysis of DFS and clinicopathological characteristics in post-mastectomy pre-cN+/ypN0 patients (n=142).

Parameters	Univariate		Multivariate	
	HR and 95%CI	P	HR and 95%CI	P
ER		0.168		0.233
Negative	Reference		Reference	
Positive	0.507(0.195-1.321)		0.520(0.177-1.525)	
HER2		0.757		0.229
Negative	Reference		Reference	
Positive	0.851(0.306-2.367)		0.507(0.167-1.536)	
Ki-67		0.475		0.344
≤15%	Reference		Reference	
>15%	1.547(0.447-5.352)		2.202(0.430-11.279)	
T		0.342		0.166
≤2cm	Reference		Reference	
>2cm	1.762(0.547-5.676)		3.051(0.631-14.766)	
Age		0.285		0.775
≤50y	Reference		Reference	
>50y	1.694(0.645-4.447)		1.178(0.384-3.614)	
RT		0.050		0.023
No	Reference		Reference	
Yes	0.356(0.127-1.000)		0.288(0.098-0.841)	

ER, estrogen receptor; HER2, human epidermal growth factor receptor2; RT, radiation treatment; HR, hazard ratio; CI, confidence interval.

## Discussion

In this retrospective analysis from two hospitals, we investigated potential clinicopathological characteristics associated with axillary response after NAT in breast cancer patients. We also studied the relationship between ypN stage and DFS. RT is usually considered essential for node-positive patients. Therefore, we explored the role of adjuvant RT in patients with different lymph node status before and after NAT.

NAT has been widely used in the treatment of early breast cancer. Overall, patients administrated with neoadjuvant and adjuvant chemotherapy showed similar long-term survival (11). Patients who achieved pCR were more likely to have a significantly better long-terms outcome (1). The response of the breast and axilla to NAT is usually highly consistent. We also observed that the axillary pCR rate varied with the breast MP grade. However, axillary pCR is only a moderate predictor of breast pCR (3, 5, 12). Therefore, the extent of axillary response and ypN stage may play different roles in predicting prognosis and guiding treatment. The HER2 amplified and triple-negative breast cancer subtypes are highly responsive to NAT and have the highest axillary pCR rate (4, 12). In our study, HER2+ breast cancer also has the highest axillary pCR rate (69.1%) and followed with TNBC (45.2%). In our cohort, about 86% of HER2+ patients received HER2-targeted therapy and achieved high axillary response rate. Anti-HER2 therapy has a vital role in improving the response rate. High Ki67 before treatment is a predictor for more pCR in neoadjuvant settings of breast cancer patients (13). Nevertheless, the cut-off value of Ki67 is still controversial. Here we find that patients with ki67 >15%+ in breast biopsy specimens are more likely to achieve axillary pCR. Further analysis indicates that Ki67 10-20%+ patients may be the most axillary responsive group. Hank Schmidt et al. (3). and Diego Flores-Funes et al. (14). reported that larger tumor size at diagnosis was associated with more residual node disease and more ALND. Our data also show similar but insignificant trend. According to our study, the younger patients show a tendency to have higher axillary pCR rate. Better performance status and compliance of the youngers may be part of the reason.

In our study, patients obtain axillary pCR from the whole cohort and initially cN+ group have significantly better DFS. However, the ypN0 and ypN1 patients have similar DFS which is different from the results of T. J. Nijnatten et al. (15). and G.C. Zhang et al. (16). Further analysis shows that about 52.8% of ypN1 patients in our cohort are categorized as Luminal A (compared with 21.7% of ypN0 cases). This indicates that Luminal A patients with cN+ at diagnosis are more likely to have limited nodal burden after NAT. J. W. Chun et al. (17) reported that the 5-year prognosis of patients with residual N1 disease who underwent sentinel lymph node biopsy (SLNB) only was similar to that of patients after ALND. The Luminal A patients may be suitable to received marker clip placement followed with selected lymph node dissection omission of ALND (18). However, more evidence is needed to certify safety. An ongoing clinical trial aims to compare ALND and axillary radiation in SLNB positive T1-3N1M0 patients treated with NAT, which may give us more message in the future (19).

Adjuvant RT had a vital role in the treatment of breast cancer with nodal metastasis. But it is still various in the clinical practice of adjuvant RT in ypN0 patients (7, 8). Y. Y. Zhang et al. (20). reported that PMRT was an independent factor to improve DFS in ypN1-3 patients. For ypN0 patients, PMRT showed no significant benefit. The results from NCDB varied. O.M. Fayanju et al. (7) indicated that adjuvant RT conferred significant OS improvement in ypN1 patients. For ypN0 cases, RT was only associated with OS benefit in post-lumpectomy patients. However, C.G. Rusthoven et al. (9) reported that PMRT could improve OS in patients with all ypN subgroups regardless of surgery type. The results of a meta-analysis suggested that PMRT might reduce local recurrence in patients with ypN0, but would not improve OS (21). In our cohort study, PMRT is an independent factor in improving DFS in pre-cN+/ypN0 patients. For ypN+ patients, adjuvant RT also shows a trend of benefiting DFS. Most of the pre-cN+/ypN0 patients in our cohort receive PMRT covering chest wall, supraclavicular and infraclavicular nodal regions (± internal mammary node). However, several studies have reported that compared with breast-only RT, regional nodal irradiation showed no extra survival improvement in ypN0 patients (7, 9, 10). Our study also has some limits. The sample size of our study is small and medium follow-up time is short, which may affect the conclusions. Besides, the no-RT post-mastectomy group has a higher fraction of HER2+ patients which is the restriction of retrospective study. The results of some ongoing randomized clinical trials may help to guide clinical practice (22).

## Conclusions

ER status, PR status, HER2 status and Ki67 are significantly correlated with ypN stage. Breast pCR is highly consistent with axillary pCR. DFS is significantly better in patients achieve axillary pCR with cN+ at diagnosis. The patients with low nodal burden (1-3 nodes) after NAT are more likely to be less aggressive subtypes and have a similar DFS with ypN0 patients. Adjuvant RT is essential in patients with initially cN+/ypN0 stage.

## Data availability statement

The raw data supporting the conclusions of this article will be made available by the authors, without undue reservation.

## Ethics statement

The studies involving human participants were reviewed and approved by Ethical Committee of the Second Affiliated Hospital of Zhejiang University. Ethical Committee of the Zhejiang Provincial People’s Hospital. Written informed consent for participation was not required for this study in accordance with the national legislation and the institutional requirements.

## Author contributions

XR, JW and QW design the work. LL, WX, RN and SW contribute the collection, analysis, or interpretation of data for the work. YY and WX contribute to the follow-up of the patients. SW helps in reviewing the histopathology slides. XR and YY write the manuscript. All authors finally approved the manuscript version to be published.
