# Supplementary material for: Axillary response and outcome in breast cancer patients after neoadjuvant treatment: The role of radiotherapy in reducing recurrence in ypN0 patients with initially cN+ stage
Source: Front Oncol. 2023 Apr 3;13:1093155. doi: 10.3389/fonc.2023.1093155 (PMC10106717; doi:10.3389/fonc.2023.1093155)
Supplement: Supplementary file 1 [file Table_1.docx]

**Table S1. The characteristics of RT and no-RT post-mastectomy patients.**

| **Characteristics** | **RT(n(%))** | **no-RT(n(%))** | **P** |  | **RT(n(%))** | **no-RT(n(%))** | **P** |
| --- | --- | --- | --- | --- | --- | --- | --- |
| **No. of patients** | 96 | 46 |  | **Grade** |  |  | 0.988 |
| **Mean age** | 51.7 | 53.5 | 0.294 | I | 3(10.0) | 2(13.3) |  |
| **ER status** |  |  | 0.292 | II | 22(73.3) | 10(66.7) |  |
| Positive | 39(40.6) | 23(50.0) |  | III | 5(16.7) | 3(20.0) |  |
| Negative | 57(59.4) | 23(50.0) |  | Unknown | 66 | 31 |  |
| **PR status** |  |  | 0.156 | **Breast response** |  |  | 0.212 |
| Positive | 40(41.7) | 25(54.3) |  | MP1 | 0(0) | 0(0) |  |
| Negative | 56(58.3) | 21(45.7) |  | MP2 | 8(8.3) | 5(10.9) |  |
| **HER2 status** |  |  | 0.011 | MP3 | 14(14.6) | 10(21.7) |  |
| Positive | 59(61.5) | 38(82.6) |  | MP4 | 16(16.7) | 8(17.4) |  |
| Negative | 37(38.5) | 8(17.4) |  | MP5 | 58(60.4) | 23(50.0) |  |
| **Ki-67** |  |  | 0.001 | **Histology** |  |  | 0.237 |
| ≤15% | 9(9.4) | 14(30.4) |  | Ductal | 89(92.7) | 39(84.8) |  |
| >15% | 87(90.6) | 32(69.6) |  | Others | 7(7.3) | 7(15.2) |  |
| **Subtypes** |  |  | 0.068 | **Axillary surgery** |  |  | 0.005 |
| HR+HER2- | 16(16.7) | 5(10.9) |  | SLNB | 0(0) | 5(10.9) |  |
| HR+HER2+ | 33(34.4) | 22(47.8) |  | ALND | 96(100.0) | 41(89.1) |  |
| HER2 amplified | 26(27.1) | 16(34.8) |  | **Neoadjuvant regimens** |  |  |  |
| TNBC | 21(21.9) | 3(6.5) |  | Anthracycline containing | 75(78.1) | 29(63.0) | 0.057 |
| **pre-cT** |  |  | 0.225 | Taxane containing | 93(96.9) | 45(97.8) | 1.000 |
| 0 | 1(1.1) | 1(2.2) |  | Single HER2 blockade | 42(43.8) | 29(63.0) | 0.031 |
| 1 | 12(13.2） | 5(11.1) |  | Dual HER2 blockade | 10(10.4) | 5(10.9) | 1.000 |
| 2 | 69(75.8) | 30(66.7) |  |  |  |  |  |
| 3 | 9(9.9) | 6(13.3) |  |  |  |  |  |
| 4 | 0(0) | 3(6.7) |  |  |  |  |  |
| Unknown | 5 | 1 |  |  |  |  |  |

RT, radiation treatment; ER, estrogen receptor; PR, progesterone receptor; HER2, human epidermal growth factor receptor2; HR, hormone receptor; TNBC, triple negative breast cancer; pre-cT, clinical tumor stage before treatment; MP, Miller-Payne; SLNB, sentinel lymph node biopsy; ALND, axillary lymph node dissection.
